# Progress in Fevers

**Published:** 1902-04-19

**Authors:** 


### PROGRESS IN FEVERS.

(*Continued from page* 25.)

**Plague.**—Further reports have been received concerning the work of the India Plague Commission.[15] As far as can be gathered from the various statistics presented by the different members of the Commission, the deaths are something like 90 per cent. greater in the uninoculated than in the inoculated. In the districts of Gadag and Betigeri the people were extremely eager to be inoculated with Haffkine's vaccine. Miss Corthorn, who has charge of the work in this district, makes the suggestion that "the people of Gadag crowded to be inoculated because they had to pay for it" (2 annas each). She considers that it is desirable at once to inoculate all who have been exposed to infection. In the new plague laboratory recently opened at Bombay[16] large quantities of the serum are manufactured. The nutritive medium is an albuminous fluid prepared from goat's flesh. This is carefully sterilised and inoculated with plague bacilli by means of a Pasteur's *ballon.* Flasks of the material so prepared are placed in the dark for six weeks, being shaken at intervals so that the colonies of bacteria formed at the surface may fall to the bottom. When no more colonies are formed the flasks are carefully heated at 65° C. and then rapidly cooled. By this means the layers of bacilli at the bottom are killed. The clear fluid above, containing their toxic products, is treated with a sterile ½ per cent. solution of carbolic acid, and syphoned into bottles which are hermetically sealed and can then be distributed for use. Throughout great care is taken to ensure asepsis. The system of harbour inspection at Bombay,[17] to prevent the spread of plague from that port, is very thorough. As soon as the cargo is unloaded the vessel is thoroughly washed with disinfectants, in case of contamination by the coolies employed in unloading, and the crew are sent on shore with the whole of their belongings and trunks. Every article is disinfected by steam, dried by hot air, and repacked in the disinfected trunk which is sealed and marked. Of the crew on shore each member undergoes careful medical inspection before returning to the ship, which he may not leave again. Each passenger also undergoes careful medical inspection before admission to the ship. All ships and boats large or small, foreign or native, are treated alike. From June 1898 to May 1899 2,708 square-rigged ships and 57,480 native craft were inspected, while 546,881 passengers and 695,136 members of crews were medically examined, of whom 15,380 passengers and 1,314 members of crews were rejected as medically unfit. Since the commencement of inspection in 1897 only seven cases of plague have occurred on ocean vessels. The work of inspection is carried out by six European medical officers, four men and two women, a large staff of subordinates, and 50 special police. The Ottoman Board of Health [18] are thinking of enforcing stringent rules compelling the destruction of rats on board plague-infected ships or those coming from infected ports. Hitherto their quarantine regulations have been very unequally and irrationally enforced.[19] In the report of the plague officials in Calcutta it is seen how virulent are the outbreaks of plague where much grain is stored and handled, and how a great mortality among rats infesting the grain is noticed previous to an outbreak.[20] The relation between rat and human plague is very plainly marked. Plague patients are sometimes found lying on sacks full of grain. In Hong Kong [21] 1,488 cases occurred during the year ending 1901. The mortality among the 26 Europeans attacked was 34·6 per cent., whilst it was 97·2 per cent. among the 1,415 Chinese attacked, the incidence and mortality being specially heavy among children. The outbreak was preceded by several weeks by a rapid rise in rat mortality. D. S. Davies [22] emphasises strongly the view that plague must be combated by destruction of rats, and quotes the dicta of Koch to that end. A plague contact is one who has been exposed to plague-stricken rats rather than plague-stricken persons. In conveyance of plague personal infection, except in the pneumonic form, plays but a small part. In Bristol, ships coming from infected ports are regarded as suspect, even if all are well on board, for the disease is often present among the rats only. There should be an international agreement whereby destruction of rats should be made compulsory at the port of departure when the hold is empty. Such an agreement has been made among the Australian Colonies, and Dr. Ashburton Thompson, of Sydney, has insisted on the need of destruction of rats in all vessels from infected ports. Farrar and Hope [23] point to infection from fleas infesting dead and living rats. The question how to destroy rats in a loaded vessel is one of great importance.[24] Sulphurous acid is most generally employed. Carbonic acid and carbonic oxide have been suggested, but are ineffective, dangerous, and impracticable. The Clayton method of disinfecting with sulphurous acid, while acknowledged by many to be effective, has been considered very costly and dangerous to many cargoes. This, however, appears to be a mistake.[25] The inventor of the method says that although, as is well known, moist goods are bleached and damaged by the gas, such things as coloured cloths and other stuffs, and comestibles such as tea, coffee, sugar, and flour are uninjured, even by prolonged and intimate exposure to a gas of 20 per cent. strength. The method has been systematically employed at New Orleans for several years, and no claim has ever been made for damage to ship or cargo. No attempt is made to so treat vessels laden with meat, fresh fruit, or liquids. One pound of sulphur supplies gas enough to saturate to the vermin-killing point 400 cubic feet of air, hence the cost is to be measured by tens instead of by the hundreds of pounds required for unloading and reloading a ship. The bacterial disinfection of the cargo is not of course effected, but rats and other vermin are thoroughly destroyed. The method consists in leading a pipe to the bottom of the hold, through which the gas is pumped, while at the same time the air at the top is withdrawn by means of an exhaust pipe.

[15] Brit. Med. Journ., Oct. 5, p. 995. [16] Lancet. Dec. 21, p. 1753. [17] Ibid., Dec. 14, p. 1692. [18] Brit. Med. Journ., Jan. 4. [19] Lancet, Nov. 30, p. 1561. [20] Brit. Med. Journ., Nov. 23, p. 1561. [21] Ibid., Nov. 23. [22] Ibid., Jan. 25, p. 207. [23] Ibid. [24] Ibid., Jan. 4, p. 30. [25] Ibid., Feb. 1, p. 294. [26] Ibid., Jan. 4, p. 30.

